# Detection of myocardial fibrosis: Where we stand

**DOI:** 10.3389/fcvm.2022.926378

**Published:** 2022-09-29

**Authors:** Leyi Zhu, Yining Wang, Shihua Zhao, Minjie Lu

**Affiliations:** ^1^State Key Laboratory of Cardiovascular Disease, Department of Magnetic Resonance Imaging, National Center for Cardiovascular Diseases, Fuwai Hospital, Beijing, China; ^2^Chinese Academy of Medical Sciences and Peking Union Medical College, Beijing, China; ^3^School of Medicine, South China University of Technology, Guangzhou, China; ^4^Key Laboratory of Cardiovascular Imaging (Cultivation), Chinese Academy of Medical Sciences, Beijing, China

**Keywords:** myocardial fibrosis, heart failure, biomarkers, non-invasive imaging, cardiovascular magnetic resonance

## Abstract

Myocardial fibrosis, resulting from the disturbance of extracellular matrix homeostasis in response to different insults, is a common and important pathological remodeling process that is associated with adverse clinical outcomes, including arrhythmia, heart failure, or even sudden cardiac death. Over the past decades, multiple non-invasive detection methods have been developed. Laboratory biomarkers can aid in both detection and risk stratification by reflecting cellular and even molecular changes in fibrotic processes, yet more evidence that validates their detection accuracy is still warranted. Different non-invasive imaging techniques have been demonstrated to not only detect myocardial fibrosis but also provide information on prognosis and management. Cardiovascular magnetic resonance (CMR) is considered as the gold standard imaging technique to non-invasively identify and quantify myocardial fibrosis with its natural ability for tissue characterization. This review summarizes the current understanding of the non-invasive detection methods of myocardial fibrosis, with the focus on different techniques and clinical applications of CMR.

## Introduction

Myocardial fibrosis is a common pathological remodeling process existed in many cardiovascular diseases, which is characterized by the excessive deposition of extracellular matrix (ECM) proteins. By causes and location, myocardial fibrosis is generally classified into two subtypes: replacement and interstitial fibrosis (1). In replacement fibrosis, the macroscopic collagen-based scar forms in response to cardiomyocyte death. Interstitial fibrosis, in contrast, represents the microscopic deposition of ECM proteins in interstitial areas, and it is not directly associated with cardiomyocyte death. Reactive fibrosis is the most common subtype of interstitial fibrosis, triggered by insults such as pressure overload. It can be seen in the course of aging and various disease conditions such as hypertension and diabetes (2). Infiltrative fibrosis is another subtype that is marked by the progressive deposition of glycosphingolipids and insoluble proteins, which can be seen in Anderson-Fabry disease and amyloidosis, respectively (3). However, replacement and interstitial fibrosis are not entirely independent. For example, myocardial infarction (MI) may induce not only replacement fibrosis in infarcted sites, but also reactive interstitial fibrosis in peri-infarct and remote areas (4).

Traditionally, myocardial fibrosis is evaluated by histopathological analysis of endocardial biopsy (EMB) or autopsy specimens, which is regarded as the gold standard to identify and quantify myocardial fibrosis. The extent of myocardial fibrosis is quantified by measuring collagen volume fraction (CVF). However, its invasive nature, together with low representativeness (failed to obtain samples of the whole myocardium) and sampling error (possible to miss localized fibrosis), limits it to become a widely accepted routine examination in clinical practice (2, 5). Over the past decades, researchers have made great efforts to develop non-invasive detection methods, including laboratory biomarkers and non-invasive imaging techniques. Cardiovascular magnetic resonance (CMR) can provide comprehensive information on myocardial anatomy, function, perfusion and tissue characterization (e.g., fibrosis, edema, fat), emerging as the non-invasive gold standard imaging technique to assess myocardial fibrosis (6). The roles of various CMR techniques to aid diagnosis, risk stratification and management have also been demonstrated (7–10). This review summarizes the current understanding of the non-invasive detection methods of myocardial fibrosis, with the focus on different techniques and clinical applications of CMR.

## ECM homeostasis: Physiology and pathology

ECM is a dynamic but tightly regulated structure, maintaining homeostasis through the synthesis and degradation of its protein components (11). This review focuses on the turnover of type I and III collagens, the primary structural proteins in ECM. In physiological conditions, fibroblasts secrete collagen precursors, called type I and III procollagens, which are then converted into mature type I and III collagens by proteinases (12). Two types of propeptides, procollagen type I carboxy-terminal propeptide (PICP) and procollagen type III amino-terminal propeptide (PIIINP), respectively coming from the carboxy-terminal of type I procollagen and the amino-terminal of type III procollagen, are released during this process and therefore can be used as the biomarkers of collagen synthesis (2, 13). Furthermore, collagens can be crosslinked by lysyl oxidases (LOXs) and LOX-like (LOXLs) enzymes to resist degradation (11). Collagen degradation is controlled by matrix metalloproteinases (MMPs, to enhance degradation) and tissue inhibitor of metalloproteinases (TIMPs, to inhibit degradation). Similarly, telopeptides such as collagen type I carboxy-terminal telopeptide (CITP) are cleaved and released during collagen degradation.

In fibrotic tissues, where different insults such as pressure overload, myocardial injury and even ischemic cardiomyocyte death occur, a series of pro-fibrotic reactions initiated by various mechanisms including neurohumoral pathways, mechanosensitive pathways, inflammatory signaling cascades and growth factors disturb the ECM homeostasis, leading to the increased collagen deposition and/or the reduced collagen degradation (2). Both activated fibroblasts and myofibroblasts can secret elevated levels of structural proteins (e.g., type I and III collagens, elastin) and pro-fibrotic non-structural proteins (e.g., fibronectin, matricellular proteins) (4, 14, 15). The circulating expression levels of some pro-fibrotic or anti-fibrotic mediators including galectin-3, soluble suppression of tumorigenicity-2 (sST2) and microRNAs (miRNAs) may change significantly during fibrotic processes; therefore, they can be used as potential biomarkers of myocardial fibrosis (13, 16). Eventually, due to the excessive ECM deposition or the loss of cardiomyocytes (in cases of MI), the extracellular space expands in fibrotic tissues.

**Figure 1:**
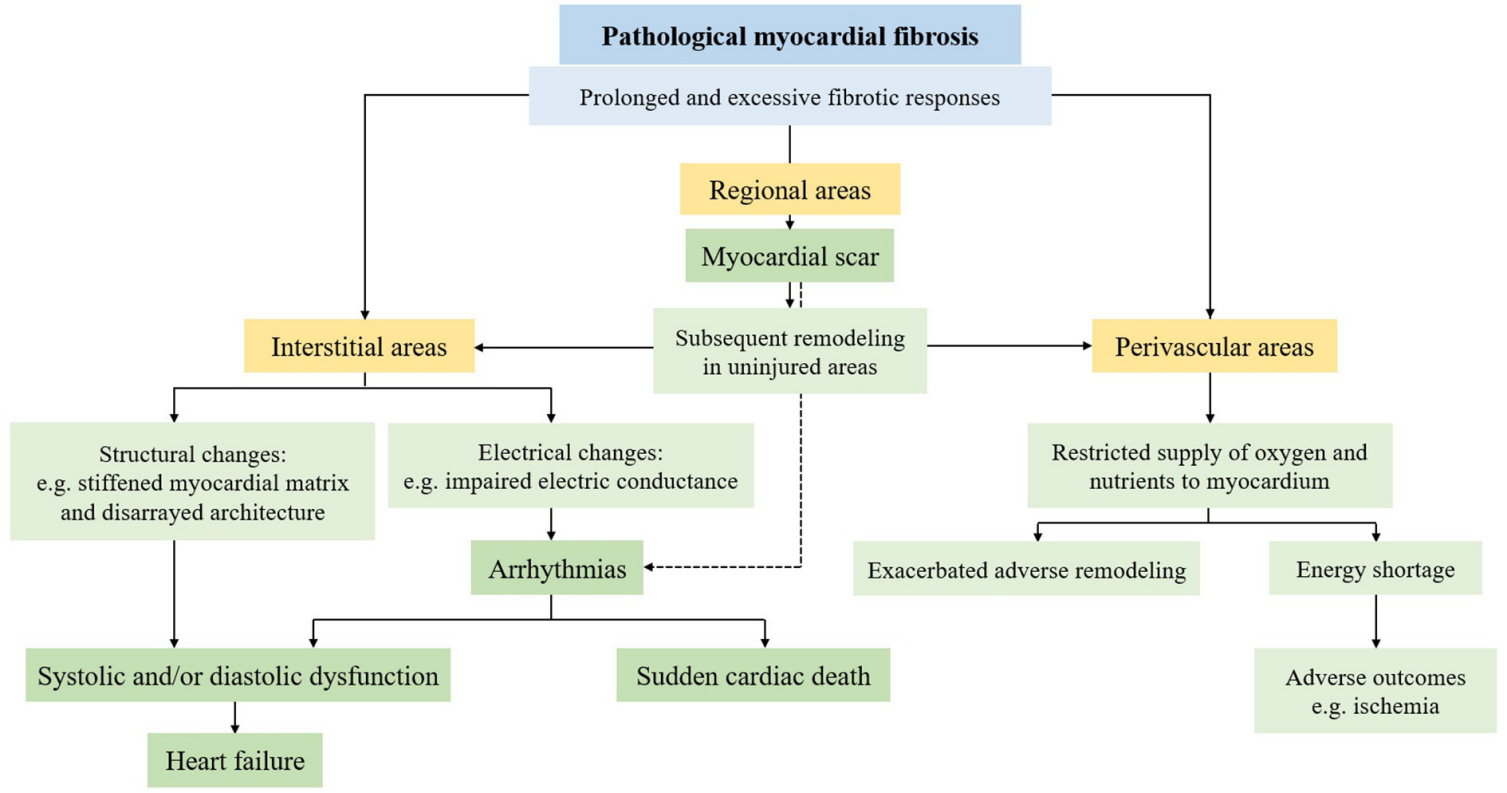
Consequences of pathological myocardial fibrosis.

Of note, the initial fibrotic process is not considered pathological because it includes protective or reparative responses against different insults. Pathological fibrosis is now appreciated as the prolonged and excessive fibrotic responses, which may result from persistent pro-fibrotic insults, or the dysregulation of pro-fibrotic factors along with the concurrent presence of myofibroblasts (17, 18). Pathological fibrosis can lead to a series of detrimental consequences (Figure 1). In interstitial fibrosis, fibrotic tissues contribute to structural and electrical changes, thus leading to alterations in cardiac function and arrhythmias. Excessive fibrosis in perivascular areas restricts the supply of oxygen and nutrients to the myocardium, which exacerbates adverse remodeling responses and makes cardiomyocytes more susceptible to adverse outcomes such as ischemia (19). In replacement fibrosis, the myocardial scar may result in the subsequent remodeling process in uninjured areas, including interstitial and perivascular fibrosis. The abnormal mechanical stress and the pro-fibrotic mediators traversing from injured areas are two underlying mechanisms of this reactive remodeling process (20, 21). It has been proposed that myocardial scar is also associated with a higher risk of ventricular arrhythmias (VAs) (22). Ultimately, some lethal cardiovascular events such as heart failure (HF) and sudden cardiac death (SCD) may occur if the excessive fibrotic responses persist.

## Detection: Laboratory biomarkers and imaging techniques

### Laboratory biomarkers

Laboratory biomarkers can be non-invasively detected *via* either the serum or plasma, thus safer compared with EMB and autopsy. They reflect cellular and even molecular changes; therefore, they can detect myocardial fibrosis in the early stage of fibrotic processes (13).

#### Collagen-derived biomarkers

PICP and PIIINP are two collagen-derived biomarkers released in the process of collagen synthesis. The levels of serum PICP and PIIINP have been shown to correlate with CVF in patients with HF (13). Both PICP and PIIINP presented high sensitivity but low specificity to identify patients with HF and preserved left ventricular ejection fraction (LVEF) (23). In HF patients with reduced LVEF (<40%), PICP presented more reliable diagnostic performance with 86.2% sensitivity and 98.8% specificity in one study (24). The prognostic value of PICP and PIIINP to predict cardiovascular and all-cause death in HF patients has also been demonstrated (25, 26). In addition, the ratio of CITP to MMP1 (CITP/MMP1) inversely correlates with the degree of collagen cross-linking. The combination of PICP and CITP/MMP1, reflecting type I collagen deposition and cross-linking respectively, is associated with a higher risk of HF rehospitalization or cardiovascular death (27), and a higher incidence or recurrence of atrial fibrillation (28). On the other hand, these collagen-derived biomarkers are not cardiac specific, and they are sensitive to comorbidities and noncardiac conditions that affect collagen turnover. Extensive validation is still needed because accumulating studies have indicated the circulating level of these biomarkers (e.g., PICP and PIIINP) did not correlate with the collagen content derived from histological examination or imaging techniques (29–31). Hence, these biomarkers have not yet been implemented in clinical practice.

#### Galectin-3 and sST2

Some novel laboratory biomarkers are associated with multiple factors including cardiac stress, inflammation and fibrosis. Galectin-3 is a beta-galactosidase-binding lectin, acting as an important inflammatory mediator in fibrotic processes. sST2 is a decoy receptor to block the cardioprotective effects of interleukin-33, which inhibits myocardial fibrosis and hypertrophy. The expression of galectin-3 and sST2 is upregulated in fibrotic processes (32, 33); however, there has been no evidence to support their correlations with CVF. Both galectin-3 and sST2 have a certain diagnostic ability to identify HF patients, and galectin-3 showed superior performance in the setting of HF with preserved LVEF (galectin-3: sensitivity 0.760, specificity 0.803; sST2: sensitivity 0.636, specificity 0.595) (34). Of note, circulating levels of galectin-3 and sST2 will increase in various inflammation-related conditions, including cancer, obesity and diabetes (35). They also lack cardiac specificity, similar to collagen-derived biomarkers. Therefore, some researchers suggested that these two biomarkers are of no value for diagnostic purposes (36). Though the diagnostic value remains uncertain, their prognostic role has been well-established. Several studies have reported the prognostic value of galectin-3 and sST2 in patients with chronic HF, and sST2 showed a superior value over galectin-3 for risk stratification (37). Furthermore, both galectin-3 and sST2 were recommended for risk stratification in the 2013 ACCF/AHA guidelines on HF (38) and its updates in 2017 (39), but they remain without evidence of an incremental management benefit (40).

#### MiRNAs

MiRNAs regulate fibrotic responses at the post-transcriptional level, and they can present and be detected in the circulation. The circulating level of miR-21, one of the most studied miRNAs, has been shown to exert fibrogenic responses (41) and positively correlate with the myocardial expression level of genes encoding type I collagen and fibronectin (42). On the other hand, several miRNAs including miR-29b and miR-203 have been suggested to be involved in the negative regulation of fibrotic processes and exert potential cardioprotective effects (43, 44). However, these miRNAs are not cardiac specific. A recent review suggested that the effect of miR-21 appears to differ, depending on various cell types and disease conditions (45). Therefore, more studies are required.

There is an increasing interest in the investigation of cardiac-specific miRNAs, which not only play a central role in cardiac development and regeneration but express abundantly in the myocardium (46). The circulating level of cardiac-specific miR-1 will increase in patients with acute MI and its overexpression can reduce fibrosis in animal models; therefore, miR-1 may serve as a novel cardiac-specific biomarker of myocardial fibrosis (46). Surprisingly, a recent study indicated that the plasma level of miR-4454, rather than miR-1, was correlated with the extent of myocardial fibrosis detected by imaging technique in patients with hypertrophic cardiomyopathy (HCM) (47). However, validation in a larger cohort is required and the origins of miR-4454 should be further researched. The cardiac-specific miR-133a has been shown to hinder myocardial fibrosis through its inhibitory effects on pathways activated by pro-fibrotic mediators such as transforming growth factor-β (TGF-β) (48, 49). In a mouse model of nonischemic dilated cardiomyopathy (DCM), miR-133a inversely correlated with several critical pro-fibrotic mediators such as fibronectin and TGF-β (48).

There are still lots of limitations of miRNAs for detecting fibrosis, including the non-specific nature of most miRNAs, the limited and sometimes controversial evidence, the suboptimal detectability of some transcripts in the circulation, and the complex and non-standardized quantification methods (16).

### Non-invasive imaging techniques

Currently, a variety of non-invasive modalities have been employed to detect myocardial fibrosis, including echocardiography, nuclear imaging, cardiac computed tomography and CMR. The role of various modalities to evaluate prognosis and aid management has also been demonstrated.

#### Echocardiography

Echocardiography is the most frequently used imaging modality for diagnosing cardiovascular diseases because of its easy availability and affordability. Fibrotic tissues may present structural changes (e.g., abnormal thickening of the myocardium) and impaired systolic and/or diastolic function in echocardiography; however, these manifestations are lack of specificity (50).

Integrated backscatter (IB) is a traditional echocardiographic approach to detect histological abnormalities of the myocardium by quantitatively assessing ultrasonic reflectivity (51, 52). There are currently two parameters of IB: the calibrated IB (cal-IB, the mean value of the IB signal calibrated by the backscatter power from the pericardium) and the cardiac-cycle-dependent variation of IB (cdv-IB) (53). Cal-IB is more frequently used as the biomarker of myocardial fibrosis and its relation with histological examination has been validated in several studies (51, 54). In one study, the area of myocardial fibrosis showed positive close correlations with cal-IB and negative close correlations with cdv-IB (53). Despite greater availability and non-invasiveness compared with EMB, the application of IB is stilled limited because IB signals are affected by various factors including structural and functional properties of the myocardium. Some validation studies showed a lack of correlation between cal-IB and myocardial fibrosis estimated by histological examination or CMR (55, 56).

The abnormal collagen deposition may affect the movement of myocardial fibers, thus leading to the adverse wall motion of the myocardium (57). Recent progress has indicated that strain parameters derived from the speckle-tracking technique can reflect the impaired myocardial deformation of fibrotic tissues. Strain parameters of both ventricles have been used to effectively detect myocardial fibrosis (58). Longitudinal strain is predominantly impaired by subendocardial fibers, and in general, it is the most sensitive deformation parameter of ventricular mechanics in cardiovascular diseases (59, 60). Therefore, global longitudinal strain (GLS) is the most studied parameter of echocardiographic strain analysis to detect myocardial fibrosis. Previous studies focused on the relation between GLS and CMR parameters, and GLS significantly correlated with the extent of fibrosis estimated by CMR in patients with HCM (61), Anderson-Fabry disease (62), and heart transplant recipients (63). Some recent studies have reported the correlations between GLS and histological fibrosis in both rat models (64) and HF patients (65). Furthermore, the prognostic value of GLS has been investigated and GLS was superior to all other echocardiographic parameters in HF patients with preserved LVEF (57, 66).

Various novel parameters incorporating data from echocardiographic strain analysis have also been introduced. Mechanical dispersion is a parameter defined as the standard deviation of time to peak negative longitudinal strain (67). In a cohort of HCM patients, mechanical dispersion correlated with the extent of fibrosis estimated by CMR, and surprisingly, it was a strong independent predictor of VAs, which is superior to GLS (68). Of note, both longitudinal strain and mechanical dispersion are not specific parameters to detect myocardial fibrosis, because they reflect the comprehensive changes of pathophysiological status in the myocardium (e.g., fibrosis, hypertrophy, microvascular ischemia) (68, 69). Myocardial work is another novel parameter of transthoracic echocardiography, reflecting the stroke work through pressure-strain loop by incorporating deformation and afterload information of the left ventricle (70). Recent studies indicated that parameters of myocardial work were associated with CMR findings of fibrosis and global constructive work (GCW) showed a better ability to predict myocardial fibrosis compared with GLS (71, 72).

However, it is critical to note that techniques of echocardiography detect fibrosis indirectly, and they are highly dependent on adequate acoustic windows.

#### Nuclear imaging

Nuclear imaging techniques, including single-photon emission computed tomography (SPECT) and positron-emission tomography (PET), can be used for detecting myocardial fibrosis. Myocardial perfusion scintigraphy is a SPECT technique where perfusion defects can indirectly reflect the myocardial scar. PET with ^15^O-labeled water (${\text{H}}_{2}^{15}\text{O}$) and carbon monoxide (C^15^O) assesses myocardial fibrosis through an indirect marker called perfusable tissue index (PTI), which represents the fraction of myocardium that is perfusable by water (73). Theoretically, PTI reduces in fibrotic tissues, where the water is not able to exchange rapidly. In a cohort of patients with chronic ischemic heart disease, PTI is inversely related to the extent of fibrosis estimated by CMR (74); while in a HCM cohort, their relation became positive due to myocardial hypertrophy and edema, both of which can expand the volume of water in fibrotic tissues and thus compensate the reduced PTI (75). Furthermore, PTI has been shown to correlate with the extent of histological fibrosis after MI in a canine model (76), yet the histologic comparisons in humans are still lacking.

Accumulating studies of nuclear medicine have paid great attention to fibrosis-specific molecular imaging. Several imaging methods, such as collagelin labeling (e.g., using ^99^mTc-labeled collagelin), activated fibroblast and myofibroblast labeling (e.g., using ^68^Ga-labeled or ^18^F-labeled fibroblast activation protein inhibitor) techniques, have emerged (6, 77, 78). Though promising, more studies are required to validate its detection accuracy in humans. Also, it is not possible to precisely delineate fibrotic regions in nuclear imaging techniques, whose spatial resolution is considerably lower than that of CMR (75). Studies examining the use of multimodality imaging (e.g., PET-CMR) for the detection of myocardial fibrosis are still ongoing.

#### CMR

CMR realizes “one-stop” inspection by assessing myocardial anatomy, function, perfusion and tissue characterization with high temporal and spatial resolution, emerging as the gold standard imaging technique to non-invasively identify and quantify myocardial fibrosis (6). There are currently two major techniques: late gadolinium enhancement (LGE) for detecting replacement fibrosis, and T1 mapping/extracellular volume fraction (ECV) for detecting interstitial fibrosis. Myocardial strain analysis, especially through CMR feature-tracking technique (CMR-FT), has shown promising to detect functional abnormalities derived from myocardial fibrosis (Table 1).

**Table 1:** Various techniques of cardiovascular magnetic resonance for detecting myocardial fibrosis.

**Technique**	**Relationship to myocardial fibrosis**	**Parameter**	**Fibrosis characterization**	**Strength**	**Limitation**
LGE	▸ Favor of detecting replacement fibrosis ▸ Correlated with the degree of histological fibrosis in human	e.g., the global and regional extent (%)	▸ Regions with high signal intensities reflect focal fibrosis/scar	▸ Widely used in clinical practice	▸ Low sensitivity to interstitial fibrosis ▸ Requires injection of GBCAs
T1 mapping/ECV	▸ Detects all types of myocardial fibrosis ▸ Correlated with the degree of histological fibrosis in human	Native T1 time (ms)	▸ Increased ▸ Needs to rule out conditions including edema and other interstitial factors	▸ Without the injection of GBCAs ▸ Relatively short scan time	▸ Reflects changes in both intracellular and extracellular space ▸ Needs robust standardization and local reference range
		Post-contrast T1 time (ms)	▸ Decreased ▸ Seldom used for detecting fibrosis	▸ Used for calculating ECV	▸ Requires injection of GBCAs ▸ Sensitive to glomerular filtration rate and other technical and physiological factors ▸ Needs robust standardization and local reference range
		ECV (%)	▸ Increased ▸ Needs to rule out conditions including edema and other interstitial factors	▸ More specific to extracellular space ▸ More stable to several technical factors	▸ Requires blood sampling to obtain hematocrit ▸ Requires injection of GBCAs ▸ Needs robust standardization and local reference range
FT	▸ Indirect ▸ Associated with the degree of histological fibrosis in human ▸ Correlated with the degree of histological fibrosis in a mouse model (79)	Global and segmental strain (%), strain rate (%/s) and rotational mechanics	▸ Impaired strain parameters reflect fibrosis	▸ Without the injection of GBCAs ▸ Fast postprocessing procedure without extra data acquisition	▸ Low reproducibility ▸ Low spatial and temporal resolution compared with echocardiography ▸ Needs further study

ECV, extracellular volume fraction; FT, feature-tracking technique; GBCAs, gadolinium-based contrast agents; LGE, late gadolinium enhancement.

##### LGE

Replacement fibrosis can be detected by late enhancement with gadolinium-based contrast agents (GBCAs). After Kim et al. initially demonstrated the association between LGE and histological replacement fibrosis in dogs (80), increasing evidence has shown their correlation in patients with various cardiovascular diseases, including HCM (81), Anderson-Fabry disease (82) and aortic stenosis (AS) (83).

LGE imaging is acquired 10–20 min after the injection of GBCAs, which does not cross membranes of cardiac cells and distributes in the extracellular space (84). Both the expansion of extracellular space and the lower clearance rate (also called the wash-out rate) can lead to the retention of GBCAs within fibrotic tissue (80, 85). With the T1 shortening effect of GBCAs, fibrotic tissue reveals with a higher signal intensity compared with the surrounding normal myocardium in T1-weighted images. Conventional LGE imaging uses an inversion recovery (IR) sequence to identify the approximate T1 of normal myocardium, with the aim to “null” the signal of normal myocardium and thus highlight the signal of fibrotic tissue (86). The selection of postprocessing methods for segmentation is crucial for detection accuracy. Manual planimetry, “n”-SD and full width half maximum (FWHM) technique are recommended by the Society for Cardiovascular Magnetic Resonance (SCMR) for standardized postprocessing (87). “n”-SD and FWHM are both threshold-based methods, where pixels with signal intensities above the threshold would be defined as fibrotic regions (88). However, the variability of signal distribution in fibrotic tissue may impact detection accuracy. For example, scars with microvascular obstruction (MVO) presenting low signal intensity in the core but high signal intensity in the border may not be defined as fibrosis in these threshold-based methods. Deep learning algorithms may provide a solution, whereas intensive studies are still needed to validate their accuracy and reproducibility.

LGE is a useful tool to differentiate between ischemic and nonischemic cardiomyopathies in clinical practice. LGE in ischemic cardiomyopathies involves the subendocardial layer corresponding to the distribution of coronary artery territories, with a variable transmural extension. LGE in nonischemic cardiomyopathies, in contrast, demonstrates nonischemic (non-subendocardial) and varied patterns. For example, patients with HCM often present LGE in the midmyocardium of right ventricular insertion points (89), while intramural LGE in basal and/or mid septum (also called “mid-wall” stripe) is often shown in patients with DCM (90). LGE in myocarditis is typically observed in the subepicardial myocardium. However, subendocardial LGE was recently reported as a severe phenotype of myocarditis, which should be differentiated from ischemic cardiomyopathies (91). Of note, LGE patterns of nonischemic cardiomyopathies mentioned above are not specific. Cumulative evidence has demonstrated the prognostic value of the presence and extent (including the global and regional extent) of LGE to predict cardiovascular death and all-cause mortality in both ischemic and nonischemic cardiomyopathies (7, 92–95). In addition to its prognostic role, LGE has been proposed for treatment decisions of cardiomyopathies. The transmural extent of LGE is employed to select pre-operative patients for revascularization because it is inversely associated with the possibility of contractile recovery (8, 96). “Gray zone” is defined as the peri-infarct areas with intermediate signal intensity between the post-infarct scar and normal myocardium (86), which may result from reactive fibrotic responses and partial volume effects (97). It is now appreciated that the comprehensive scar evaluation including post-infarct scar and surrounding “gray zone” is more associated with VAs and SCD than LVEF, the current selection criteria for implantable cardioverter-defibrillators therapy (22, 98). Similarly, LGE has a stronger prognostic value for SCD risk stratification than LVEF in nonischemic cardiomyopathies, especially for patients with LVEF>35% (99, 100).

Intensive exercise may induce a series of remodeling responses in cardiovascular structure and function, including myocardial fibrosis. Increasing studies have demonstrated that some athletes could develop cardiac remodeling that may overlap with features of mild forms of cardiomyopathy, known as the diagnostic “gray zone” (101). CMR is a feasible approach to differentiate these physiological adaptations from pathological cardiomyopathies with its abilities of tissue characterization and quantitative measurement. In endurance athletes, inferior right ventricular insertion points LGE is the most common pattern, which represents the microinjuries in regions of tension concentration (102, 103). Other differential features include the symmetric and mild dilation of four chambers, the balanced and concentrically increased wall thickness, and the enhanced bilateral stroke volumes (102, 104).

Valvular heart diseases involve not only valve lesions, but also the remodeling process in the myocardium, including fibrotic responses. There are two types of myocardial fibrosis in patients with AS: interstitial fibrosis against pressure overload developed in the early stage of the disease process, and replacement fibrosis in the midmyocardium developed along with disease progression (105). The latter can be detected in LGE imaging and it is an important predictor of cardiovascular and all-cause mortality (106).

LGE plays a vital role in both diagnosis and risk stratification for infiltrative diseases. The typical LGE pattern of cardiac amyloidosis is diffuse subendocardial or transmural, which provides not only high sensitivity and specificity for diagnosis but also a strong prognostic value for all-cause mortality (107–109). Myocardial fibrosis can be the first sign of organ involvement in patients with Anderson-Fabry disease (110) and it typically presents LGE in the basal inferolateral myocardium (111).

Of note, standardization must be performed because parameters of LGE can be influenced by different factors such as field of strength, amount of GBCAs, type of sequences and segmentation methods (1). The low sensitivity for detecting interstitial fibrosis due to its comparative nature, and the low spatial resolution due to respiratory motions as well as heart rate variabilities are two major limitations of LGE (88). Despite the extended scan time, the novel three-dimensional high-resolution LGE technique may be a promising approach.

##### T1 mapping and ECV

T1 mapping and ECV technique provide not only insights into interstitial fibrosis, but also the quantitative evaluation of tissue characterization based on fully quantitative pixel-wise analysis. T1 mapping parameters (including native T1, post-contrast T1 and ECV) have been demonstrated to correlate with the degree of histological interstitial fibrosis (112), and ECV has exhibited excellent agreement with extracellular space derived from histological examination (113).

Theoretically, each voxel of the myocardium can be measured on T1 mapping images. Based on IR, the modified Look-Locker inversion-recovery (MOLLI) is the most widely-used pre-pulse sequence, using multiple Look-Locker sequences at the same phase of cardiac cycle within one breath hold (1, 114). After each Look-Locker pre-pulse, the readout sequence (e.g., steady-state free precession) acquires signals following an exponential course, and identifies the exact T1 value through a curve-fitting process (6). Limitations of MOLLI are the long breath-hold time and the sensitivity to heart rate. To date, novel sequences have emerged, such as short MOLLI and saturation pulse-prepared heart rate-independent IR (SASHA). T1 map is a reconstructed image after postprocessing, where the T1 value of each voxel is encoded as the signal intensity (or color) of each corresponding pixel.

Of note, either native T1 or post-contrast T1 is influenced by changes in both intracellular and extracellular space (1). ECV is a more specific parameter targeting extracellular space, and it is calculated by incorporating parameters of native and post-contrast T1 and correcting the effect of hematocrit (115). Moreover, ECV is relatively stable in contrast to native T1, because it is a ratio parameter offsetting the influence of several technical factors such as field of strength, imaging sequences and GBCAs. Increased native T1 and ECV can be seen in myocardial edema (e.g., in acute MI or myocarditis) or expansion of extracellular space (e.g., in myocardial fibrosis or infiltrative diseases). ECV is considered as the most sensitive marker of myocardial fibrosis after ruling out edema and other interstitial factors such as infiltrative diseases (113). However, the requirement of blood sampling for hematocrit at the time of scanning limits the routine use of ECV qualification. Based on the linear relationship between the hematocrit and the native T1 of blood, synthetic ECV is a new method to determine ECV without blood sampling (116). Recently, a large cohort study showed synthetic ECV presented no statistical difference from conventional ECV estimated by CMR and measured ECV by histological examination (117).

**Figure 2:**
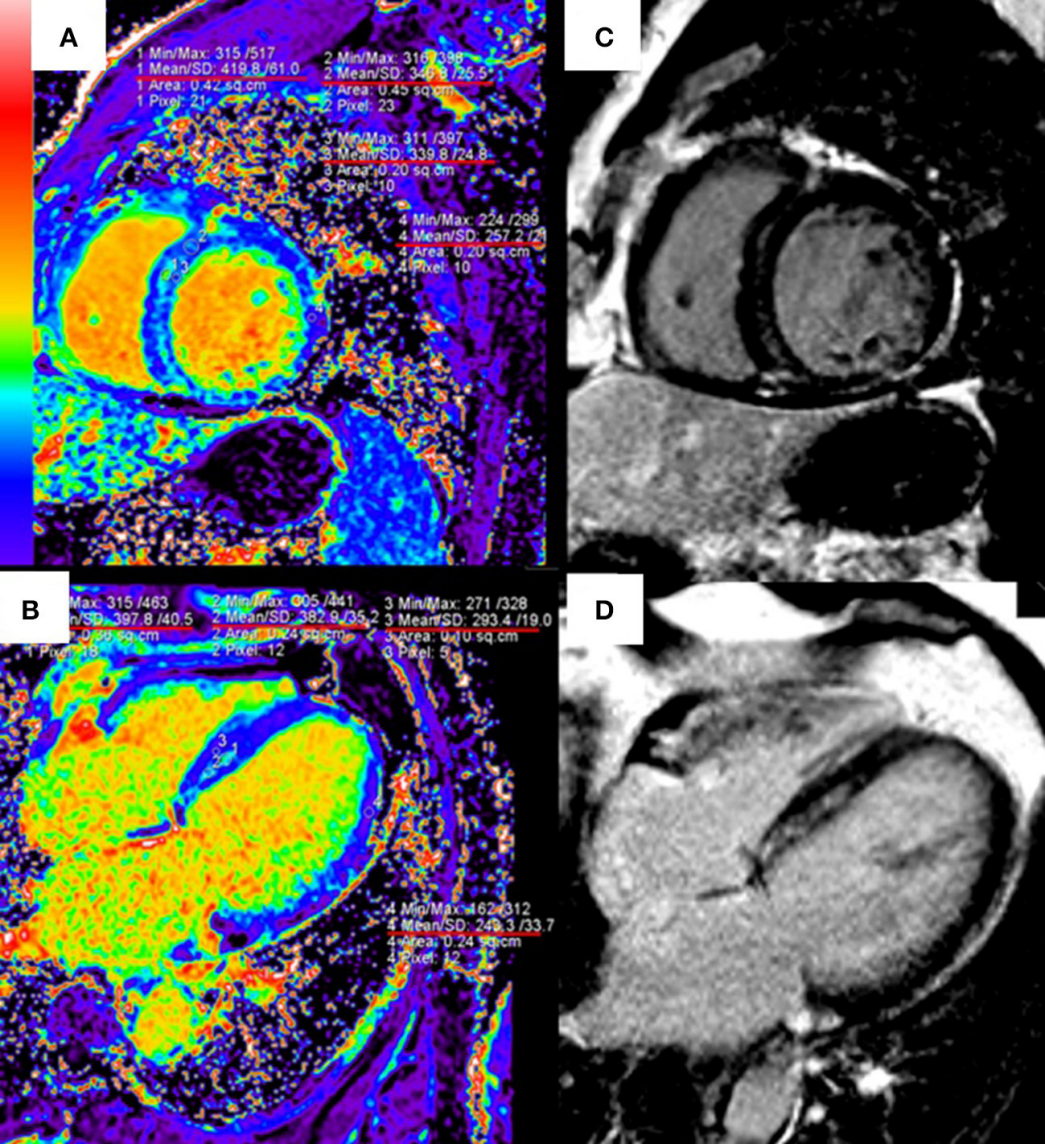
Quantitative extracellular volume (ECV) fraction images [left column, **(A,B)**] and late gadolinium enhancement (LGE) [right column, **(C,D)**] in a hypertension patient. The abnormal ECV region is larger than LGE. Reprinted with permission from Wang et al. (120).

T1 mapping and ECV are useful to quantify the infarct size and the extent of “gray zone” in ischemic cardiomyopathies. The early CMR imaging with T1 mapping and ECV is also a potent diagnostic tool for identifying the etiology of MI with non-obstructive coronary arteries (118). In patients with nonischemic cardiomyopathies such as HCM and DCM, native T1 and ECV have shown strong diagnostic and prognostic values, especially for patients without LGE (9, 119). ECV can identify diffuse myocardial fibrosis at the early stage of hypertensive cardiomyopathy, before the appearance of LGE (120) (Figure 2). T1 mapping is also included in the updated Lake Louise Criteria (CMR consensus recommendations for myocarditis) in 2018 to detect edema and reactive fibrosis during the early inflammatory phases (121) and it can significantly improve the diagnostic ability of CMR for acute myocarditis (122).

The introduction of ECV provides new differential features between exercise-induced remodeling and pathological cardiomyopathy. The left ventricular mass increases during long-term intensive exercise, thus leading to the expansion of intracellular space and the relative decrease of ECV (123). Therefore, as most studies have demonstrated, regions of myocardial fibrosis in athletes present a decreased or normal ECV, while a high ECV is generally observed in cardiomyopathies (102, 124).

T1 mapping and ECV are particularly useful to detect myocardial fibrosis in infiltrative interstitial diseases. Native T1 significantly increases in patients with cardiac amyloidosis, providing high accuracy for diagnosis (125). In contrast, native T1 decreases in patients with Anderson-Fabry disease. It has been revealed that the low native T1 can occur earlier than the appearance of left ventricular hypertrophy, which is the most common CMR finding in Anderson-Fabry disease (126).

It should be pointed out that some conditions can result in the reduction of native T1 (e.g., excessive deposition of fat or iron) and ECV (e.g., myocardial hypertrophy). For example, false-negative results of fibrosis may occur in cases of MI with MVO (vascular blockage) or intramyocardial hemorrhage (containing methemoglobin), or in cases of Anderson-Fabry disease (fat deposition) (6). Parameters in T1 mapping and ECV technique are still dependent on various technical and physiological factors; therefore, both standardization and local reference range are needed (127).

##### Myocardial strain analysis

Unlike LGE and T1 mapping, strain analysis detects myocardial fibrosis indirectly by assessing the mechanics and deformation of the myocardium without contrast agents. Tagging technique is a traditional tool for strain analysis; however, its use is limited by complex postprocessing procedures. CMR-FT is a more feasible approach based on the fast postprocessing of cine images, acquiring strain information (e.g., strain, strain rate, rotational mechanics) globally or segmentally in three orthogonal directions: longitudinal, circumferential and radial (50). Multiple strain parameters have been demonstrated their association with CMR tagging (128), LGE (129) and histological examination (130).

The diagnostic ability of strain parameters derived from CMR-FT has been evaluated in recent years. Global and segmental strain could respectively stratify different extent and transmurality of LGE in patients with ST-segment elevation MI, and the surrounding areas of the infarcted core without LGE had impaired strains (129). Segmental rather than global longitudinal strain, especially septal longitudinal strain, was associated with histological fibrosis in HCM and provided incremental values over LGE to detect myocardial fibrosis (130, 131). In addition, the value of CMR strain parameters for risk stratification has also been reported (10, 132).

However, the reproducibility and spatial resolution of CMR-FT are relatively low compared with echocardiography.

## Conclusions and future perspectives

The excessive ECM deposition resulting from disturbed ECM homeostasis is the major pathophysiology of myocardial fibrosis, a pathological remodeling process associated with a poor prognosis. Different non-invasive detection methods have been employed over the past decades. Laboratory biomarkers begin to take shape from diagnosis to risk stratification, and miRNAs seem promising, yet the evidence remains limited and sometimes mixed. Significant progress has been made in non-invasive imaging techniques, among which CMR is considered as the non-invasive gold standard imaging technique to identify and quantify myocardial fibrosis with its natural ability for tissue characterization, providing comprehensive information on diagnosis, prognosis, risk stratification and management.

Studies examining the combination strategy of laboratory biomarkers and imaging techniques to increase diagnostic accuracy and improve risk stratification are currently under investigation. The combination of LGE and circulating biomarkers including PICP and galectin-3 has been shown an additive prognostic value in nonischemic cardiomyopathies (133, 134). Yet researches regarding the diagnostic role of this combination strategy are still limited. Furthermore, there is also no well-established non-invasive detection method targeting myocardial fibrosis specifically. Both collagen- and elastin-specific CMR contrast agents have been developed, bringing hope for the direct quantification of interstitial spaces (135, 136). Hopefully, future research on detection methods of myocardial fibrosis will help, to detect diseases with higher accuracy, to allow earlier diagnosis of diseases, and ultimately to reduce morbidity and mortality.

## Author contributions

ML conceived this idea and was in charge of the overall responsibility of this work. LZ drafted the manuscript, which was revised by YW, SZ, and ML. All authors reviewed and approved the submitted version.

## Funding

This work was supported by the Construction Research Project of the Key Laboratory (Cultivation) of Chinese Academy of Medical Sciences (2019PT310025), the National Natural Science Foundation of China (81971588), the Youth Key Program of High-level Hospital Clinical Research (2022-GSP-QZ-5), the Capital Health Research and Development of Special (2020-2-4034), and the Clinical and Translational Fund of Chinese Academy of Medical Sciences (2019XK320063).

## Conflict of interest

The authors declare that the research was conducted in the absence of any commercial or financial relationships that could be construed as a potential conflict of interest.

## Publisher's note

All claims expressed in this article are solely those of the authors and do not necessarily represent those of their affiliated organizations, or those of the publisher, the editors and the reviewers. Any product that may be evaluated in this article, or claim that may be made by its manufacturer, is not guaranteed or endorsed by the publisher.
